# Tuberculosis caused by *Mycobacterium bovis* infection in a captive-bred American bullfrog (*Lithobates catesbeiana*)

**DOI:** 10.1186/s12917-018-1618-6

**Published:** 2018-09-21

**Authors:** Cassia Yumi Ikuta, Laura Reisfeld, Bruna Silvatti, Fernanda Auciello Salvagni, Catia Dejuste de Paula, Allan Patrick Pessier, José Luiz Catão-Dias, José Soares Ferreira Neto

**Affiliations:** 1 Aquário de São Paulo, Rua Huet Bacelar 407, São Paulo, SP 04275-000 Brazil; 2 Universidade de São Paulo, Faculdade de Medicina Veterinária e Zootecnia, Avenida Prof. Dr. Orlando marques de Paiva 87, São Paulo, SP 05508-270 Brazil; 3 College of Veterinary Medicine, Washington State University, Pullman, WA 99164-7040 USA

**Keywords:** Amphibian, Bovine tuberculosis, Bullfrog, *Mycobacterium bovis*

## Abstract

**Background:**

Tuberculosis is widely known as a progressive disease that affects endothermic animals, leading to death and/or economical losses, while mycobacterial infections in amphibians are commonly due to nontuberculous mycobacteria. To the authors’ knowledge, this report describes the first case of bovine tuberculosis in a poikilothermic animal.

**Case presentation:**

An adult female captive American bullfrog (*Lithobates catesbeianus* Shaw, 1802) died in a Brazilian aquarium. Multiple granulomas with acid-fast bacilli were observed in several organs. Identification of *Mycobacterium bovis* was accomplished by culture and PCR methods. The other animals from the same enclosure were euthanized, but no evidence of mycobacterial infection was observed.

**Conclusions:**

The American bullfrog was introduced in several countries around the world as an alternative husbandry, and its production is purposed for zoological and aquarium collections, biomedical research, education, human consumption and pet market. The present report warns about an episode of bovine tuberculosis in an amphibian, therefore further studies are necessary to define this frog species’ role in the epidemiology of *M. bovis*.

## Background

The genus *Mycobacterium* comprises several species, such as members of the *Mycobacterium tuberculosis* complex (MTBC) and other species known as nontuberculous mycobacteria (NTM) [1]. *Mycobacterium bovis* belongs to the MTBC, and it is well-known for causing bovine tuberculosis and for its wide host range, including humans [1, 2]. There are reports of *M. bovis* infection in many species of mammals [3], while they are rare in birds [4].

In amphibians, mycobacterial infections are commonly caused by NTM, such as *M. marinum*, *M. chelonae*, *M. fortuitum* and *M. ulcerans* ecovar Liflandii, and are a common source of morbidity and mortality in captive animals [5]. Natural transmission is still poorly understood, but most NTM infections in amphibians are thought to be opportunistic and acquired from environmental sources, such as soil, water and biofilms [5, 6].

International amphibians trade of captive-bred and wild-caught individuals has connected continents, as they are intended for zoological and aquarium collections, biomedical research, education [7, 8], human consumption [8, 9] and pet market [5, 7]. An example of a heavily traded amphibian is the American bullfrog (*Lithobates catesbeianus* Shaw, 1802). This species has been introduced in Central and South America, the Caribbean islands and Northeast and South-eastern Asia from North America, and it has been farmed for food, pet and biomedical research in many regions around the world [10, 11].

This report describes a case of tuberculosis caused by *Mycobacterium bovis* in a specimen of *Lithobates catesbeianus* kept in an aquarium and the potential zoonotic risk to the animal handlers.

## Case presentation

A female American bullfrog (*Lithobates catesbeianus*) was acquired from a frog farm in the state of Sao Paulo that provided animals for meat, research institutes and schools. The animal was transferred to a public aquarium in Brazil, where it was maintained in a closed aqua-terrarium provided with adequate humidity, temperature and water quality. In the same enclosure, exclusive for bullfrogs, there were other three specimens. All of them were previously kept in quarantine for 30 days.

After five years in captivity, with no previous health problems, the animal showed an episode of regurgitation of a bloody fluid. During physical examination, anorexia and pale mucous membranes were observed and the animal was in shock. Despite attempt of supportive treatment with dexamethasone (1 mg/kg), enrofloxacin (1.5 mg/kg), and mixed amino acids and B-vitamins (0.5 ml, Mercepton®) by intramuscular administration, the animal died after 2 h.

At necropsy, 5 ml of translucent viscous fluid was observed in the coelomic cavity, and on cytological evaluation it showed a large amount of proteinaceous material, small numbers of erythrocytes, rare macrophages, lymphocytes and plasma cells, cellular debris and squamous epithelial cells. Macroscopically, isolated or agglomerated, 1.0 to 3.0 mm diameter, firm white-yellow nodules with caseous content were observed in the heart, lungs, liver, spleen, stomach, intestine, gonadal fat bodies and kidneys. The nodules were most frequent in the liver and spleen, and the spleen was firm and 3 to 4 times normal size.

Tissues samples from all organs were placed in 10% buffered formalin, and five-micrometer sections were obtained and stained using hematoxylin-eosin and Ziehl-Neelsen techniques. Microscopically, the lungs, spleen, intestine, liver, pancreas and kidneys had multiple granulomas, and large numbers of acid-fast bacilli (AFB) within macrophages consistent with a mycobacterial infection. Diffuse congestion, multifocal hemorrhage, extensive necrotic areas were observed in all organs, and marked hyperplasia of melanomacrophage centers were noted in spleen and liver.

Swab samples from all affected organs and the regurgitated bloody fluid were treated with 0.75% 1-Hexadecylpyridinium chloride (HPC), cultured in Stonebrink and Löwenstein-Jensen media, and incubated at 25 °C and 37 °C for 90 days. After approximately 35 days, mycobacterial isolates were observed in Stonebrink medium at 37 °C from all samples, and identified as *Mycobacterium* sp., MTBC, and *M. bovis* through previously described PCR methods, based on 16S rRNA gene, MPB70 gene [12] and genomic regions of difference (RD) [13], respectively.

Direct Variable Repeat Spacer Oligonucleotide Typing (DVR-Spoligotyping) [14] and the 24-locus set of mycobacterial interspersed repetitive-unit-variable-number tandem-repeat (MIRU-VNTR) [15] techniques were performed for genotyping. The *M. bovis* isolates were discriminated as SB2444 by the Mycobacterium bovis Spoligotyping Database (https://www.mbovis.org) and presented the unique pattern 2 2 5 3 2 2 3 3 2 2 6 3 4 4 4 0 5 3 3 3 2 7 1 2 through the MIRU-VNTR plus database (https://www.miru-vntrplus.org/MIRU/index.faces).

Despite the lack of clinical signs, the other animals in the enclosure were euthanized. They were firstly anesthetized with ketamine hydrochloride (100 mg/kg) and xylazine (10 mg/kg) by intracelomic administration (IC), followed by a lethal dose of pentobarbital sodium (60 mg/kg), IC. Necropsy, histopathological and bacterial examinations were performed, but no evidence of mycobacterial infection was observed.

## Discussion and conclusions

Aquaculture of American bullfrogs for the meat industry has expanded worldwide during the twentieth century [10]. Taiwan, Brazil, Ecuador and China are known for their significant production [8], while the United States of America, France, Canada, Belgium, Italy and Spain are known as great consumers [10]. Brazil is the second largest producer of American bullfrogs in the world at approximately 400 tons per year [16]. The success of the industry is attributable to the advantageous Brazilian climate and improvement of production systems that were expanded in the 1970’s and improved with the assistance of research institutions and universities [9], to provide controlled conditions for better efficiency.

To the authors’ knowledge, the present study describes the first report of infection by a member of the MTBC in a poikilothermic animal. Given the optimal growing temperature of MTBC is 37 °C, it is odd that a frog was able to develop bovine tuberculosis. However, the Brazilian climate can reach over 30 °C during winter, and higher temperatures are kept through climatized production systems.

Moreover, this bullfrog could be immunocompromised, considering the other animals showed no signs of infection. Similar disseminated granulomatous lesions caused by *M. marinum* were observed in American bullfrogs [17] and Japanese forest green tree frogs (*Rhacophorus arboreus*) [18]. The coelomic effusion seen in the latter [18] and in the specimen of this case resembles the ascitic fluid caused by *M. tuberculosis* and *M. bovis* in humans [19]. Accordingly, it is not surprising the use of *M. marinum* infection in fish and frogs as model for human tuberculosis [18].

The source of the *M. bovis* infection in this bullfrog was not determined. The aquarium handlers were tested for tuberculosis and presented negative results. Transmission by visitors is unlikely, since there is a glass barrier avoiding any contact to them. Also, each enclosure is isolated from the other and from any external contact, thus the infection probably occurred in the frog farm. Unfortunately, the information regarding the farm was lost during the aquarium personnel change.

Despite improvement of raniculture production systems, not all farms have adopted best management practices and some of them may supply the animals with crude protein feed from different sources. Given *L. catesbeianus* becomes carnivore in its adult stage [9, 10], our specimen might have acquired the infection from contaminated cow meat fed to the frog while in the farm. Other transmission possibilities could be through contact with an infected animal, whether from wildlife or another domestic animal source. Finally, an environmental source of infection cannot be excluded because while *M. bovis* is an obligate pathogen [1], it can survive in the environment for weeks depending on temperature, sunlight exposure, soil moisture and the presence of organic matter [20].

Although the genotyping techniques for bovine tuberculosis are purposed to track the origin of the infection, the profile found has not been reported in any study. Yet, it resembles the three most common spoligotypes in Brazil, SB0295, SB0121 and SB0120 [21], displaying two, three and four different spacers, respectively.

Isolation of *M. bovis* in captive American bullfrogs, either in breeding or research facilities and zoo collections, raises concerns for a new potential source of this highly zoonotic disease to workers in direct contact with these animals and for international movement as the result of trade in frog meat. Furthermore, the American bullfrog is ranked as one of the world’s most invasive species by the International Union for Conservation of Nature (IUCN) Invasive Species Specialist Group (ISSG) [22] and escape or release of frogs has resulted in feral populations [23].

Nevertheless, this case reports one episode of bovine tuberculosis in an amphibian, and further studies with experimental data are necessary to define if this frog species represents a potential reservoir or acts as spillover host of *M. bovis*.
